# Effect of risedronate on joint structure and symptoms of knee osteoarthritis: results of the BRISK randomized, controlled trial [ISRCTN01928173]

**DOI:** 10.1186/ar1716

**Published:** 2005-03-24

**Authors:** Tim D Spector, Philip G Conaghan, J Christopher Buckland-Wright, Patrick Garnero, Gary A Cline, John F Beary, David J Valent, Joan M Meyer

**Affiliations:** 1 Twin Research & Genetic Epidemiology Unit, St Thomas' Hospital, London, UK; 2 Academic Unit of Musculoskeletal Disease, University of Leeds, UK; 3 King's College London, School of Biomedical Sciences, London, UK; 4 SYNARC, Lyon, France; 5 Procter & Gamble Pharmaceuticals, Mason, OH, USA

## Abstract

To determine the efficacy and safety of risedronate in patients with knee osteoarthritis (OA), the British study of risedronate in structure and symptoms of knee OA (BRISK), a 1-year prospective, double-blind, placebo-controlled study, enrolled patients (40–80 years of age) with mild to moderate OA of the medial compartment of the knee. The primary aims were to detect differences in symptoms and function. Patients were randomized to once-daily risedronate (5 mg or 15 mg) or placebo. Radiographs were taken at baseline and 1 year for assessment of joint-space width using a standardized radiographic method with fluoroscopic positioning of the joint. Pain, function, and stiffness were assessed using the Western Ontario and McMaster Universities (WOMAC) OA index. The patient global assessment and use of walking aids were measured and bone and cartilage markers were assessed. The intention-to-treat population consisted of 284 patients. Those receiving risedronate at 15 mg showed improvement of the WOMAC index, particularly of physical function, significant improvement of the patient global assessment (*P *< 0.001), and decreased use of walking aids relative to patients receiving the placebo (*P *= 0.009). A trend towards attenuation of joint-space narrowing was observed in the group receiving 15 mg risedronate. Eight percent (*n *= 7) of patients receiving placebo and 4% (*n *= 4) of patients receiving 5 mg risedronate exhibited detectable progression of disease (joint-space width ≥ 25% or ≥ 0.75 mm) versus 1% (*n *= 1) of patients receiving 15 mg risedronate (*P *= 0.067). Risedronate (15 mg) significantly reduced markers of cartilage degradation and bone resorption. Both doses of risedronate were well tolerated. In this study, clear trends towards improvement were observed in both joint structure and symptoms in patients with primary knee OA treated with risedronate.

## Introduction

Osteoarthritis (OA) is a chronic, progressive disease that particularly affects weight-bearing joints such as hips and knees. The entire joint is affected by a complex combination of degradative and reparative processes, which alter the anatomy and function of articular cartilage, subchondral bone, and other joint tissues. Of the joints affected, knee OA in particular is a major cause of morbidity, often resulting in knee replacement [1-3]. Moreover, costs associated with OA are high – in the USA alone in 1991, the annual cost of knee replacements was estimated to be more than one billion dollars [4]. OA is normally the result of an interplay between systemic (e.g. age, obesity) and local (e.g. sports injury) factors that affect the joints of the body. Radiographic evidence has shown that joint-space narrowing (a surrogate marker for articular cartilage [5]), sclerosis of the subchondral bone, and the presence of osteophytes are typical structural features of OA.

Current therapies for OA are largely aimed at providing symptom relief, and include a wide range of analgesics (e.g. nonsteroidal anti-inflammatory drugs and cyclooxygenase-2 agents). In contrast, only limited data are available about therapies that modify the course of the disease or affect joint structure. Historically, OA has been considered a disease of the cartilage, but more recent evidence suggests that subchrondral bone is also involved in the pathogenesis, in both disease initiation and progression. For example, increased local bone turnover, decreased bone mineral content and stiffness, and decreased trabecular numbers have been observed in OA subchondral bone structure compared with normal bone [6-8]. There is an increased level of interest in subchondral bone as a therapeutic OA target, and, in particular, the possibility that drugs affecting bone metabolism might alter the progression of knee and hip OA.

The Duncan-Hartley guinea pig model is a widely used spontaneous model of OA progression [9]. Several recent OA studies have evaluated this model for the effects of the antiresorptive agents bisphosphonates. For example, a comparative analysis of multiple bisphosphonates showed that only the group of nitrogen-containing bisphosphonates with pyridinyl sidechains demonstrated significant effects on the cartilage, although not all of these proved effective [10]. In a separate study using the guinea pig OA model, the pyridinyl bisphosphonate risedronate was shown to slow disease progression, as measured by the size and severity of cartilage lesions and the size of osteophytes, by up to 40% [11]. Based upon these preclinical studies, a clinical trial was performed in order to evaluate the effects of risedronate in patients with mild to moderate knee OA. The primary end points were changes in symptoms and function, with secondary end points of changes in joint structure or in markers of joint structure.

## Materials and methods

### Study design and selection of patients

The British Study of Risedronate in Structure and Symptoms of Knee OA (BRISK) was a 1-year prospective, double-blind, placebo-controlled study conducted in 10 centres in the UK. Male and female subjects aged 40 to 80 years with mild to moderate medial-compartment knee OA, diagnosed according to the clinical and radiological criteria of the American College of Rheumatology [12], were recruited into the trial.

OA in at least one knee, designated the signal knee, was required to meet the following clinical and radiographic inclusion criteria. Clinical inclusion criteria were the presence of daily knee pain for at least 1 month out of the 3 months preceding the study, with at least one of the following: age >50 years, morning knee stiffness of <30 minutes, or knee crepitus. The radiographic criteria for inclusion were a joint-space width (JSW) of 2 to 4 mm in the medial tibiofemoral compartment in the semiflexed anterior–posterior (AP) view of the signal knee and a narrower width than in the lateral compartment of the same knee. Patients were also required to have at least one osteophyte in either the medial or the lateral compartment of the tibiofemoral joint. Major exclusion criteria were the presence of rheumatic diseases that could be responsible for secondary OA; use of intra-articular hyaluronic acid in the signal knee; knee injury or diagnostic arthroscopy of the signal knee in the 6 months preceding enrollment; a history of knee surgery (including arthroscopy requiring an incision of internal joint components) in the signal knee at any time; intra-articular corticosteroids in the 3 months preceding enrollment; the presence of non-OA causes of knee pain in the signal knee (e.g. anserine bursitis, fibromyalgia, or osteonecrosis); and the use of bisphosphonates within the 12 months preceding enrollment.

The subjects gave their written, informed consent before entering the study, which was conducted in accordance with the International Conference on Harmonization (ICH) guidelines for Good Clinical Practice (GCP) and was approved by the UK Multicentre Research Ethical Committee (MREC).

### Treatment assignment

The subjects were randomly assigned in a 1:1:1 ratio to one of three arms to receive risedronate at 5 mg or 15 mg or placebo once daily for 1 year. Before randomization, patients were stratified according to their current use of oestrogen or a selective oestrogen receptor modulator.

The subjects were instructed to take their study medication with at least 120 mL of water, 30 minutes before breakfast, or, if the medication was taken later in the day, at least 2 hours before or after food intake and at least 30 minutes before bedtime. They were instructed to take their study medication while they were upright and not to lie down for at least 30 minutes afterwards.

### Symptom outcome measures

The outcome instrument for evaluation of risedronate efficacy on symptoms of OA was the Western Ontario and McMaster Universities (WOMAC) OA index [13]. The visual analogue scale (VAS) of the index was used, in which patients assessed each question using a 100-mm scale, with a higher score representing greater symptom severity. The total index score for the signal knee corresponded to the weighted composite of the 24 question scores standardized to a 100-point scale. Scores were also determined for the subscales of pain (5 questions), stiffness (2 questions), and physical function (17 questions). Other symptom outcome measures included the patient global assessment (PGA) of disease, consumption of pain medication, and the use of walking aids. For the PGA, patients answered the following question using a VAS: "Considering all the ways your OA affects you, how have you been in the last 48 hours?" Patients marked values on a scale from 0 to 100 mm.

A step-down reduction in the use of pain medication was effected 5 days before all symptom evaluations. Patients were provided with approximately 30 tablets each of paracetamol (500 mg) and diclofenac (50 mg) to be used as the only pain medication 3 to 5 days before the baseline assessment and at visits at months 3, 6, and 12 (the exit visit). No pain medications were to be used 2 days before the scheduled evaluation date or on the day itself, with the exception of low-dose acetylsalicylic acid (<350 mg/day) for cardiac protection. Rescue analgesia was permitted during the study except for the 2-day washout period before each visit.

### Structure outcome measures

The outcome measure for assessment of joint structural changes was the mean change from baseline values in minimum JSW of the medial compartment of the knee. Radiographs of the knee were taken at baseline and at 1 year using a standardized radiographic method with fluoroscopic positioning of the joint in a semiflexed position [14,15]. By the use of this technique, the anterior and posterior rims of the tibia were aligned (to within 1 mm) for reproducible positioning. Radiographs were subjected to extensive quality control at the radiographic facility before dispatch to the Central Analysis Center [15]. Radiographs were read centrally and their quality control was rechecked before computer software was used to obtain the radiographic magnification. This was determined from measurement of a metal ball placed at the head of the fibula at the time of radiography and was used to adjust the computerized measurement obtained of the minimum medial compartment JSW [15]. The test–retest standard deviation of the difference between radiographs taken 2 days apart for this technique was approximately 0.2 mm, based upon repeat measurements in 199 subjects [15]. A retrospective analysis was performed taking into account the precision of the instrument. Retrospectively, clinically meaningful disease progression was defined as joint-space narrowing of ≥ 0.75 mm or a ≥ 25% loss from baseline values. The ≥ 0.75-mm value is almost four times the 0.2-mm standard deviation observed for the x-ray method.

### Structure–symptom relation

The relation between knee OA symptoms and radiographic joint-space narrowing was assessed retrospectively; the mean change in symptom scores between baseline to month 12 of the total WOMAC score and pain and function subscales was compared with the magnitude of change in JSW over the study period.

### Bone and cartilage markers

Early-morning fasting urine and serum samples were collected at baseline and at months 3, 6, and 12 for assessment of markers of bone and cartilage turnover. Bone resorption was assessed by measurement of urinary levels of the N-terminal crosslinking telopeptide of type I collagen (NTX-I, Osteomark; OrthoClinical Diagnostics, High Wycombe, Bucks, UK) [16]. Bone formation was assessed by measurement of bone-specific serum alkaline phosphatase (Ostase, Beckman-Coulter, San Diego, CA, USA) [17] and cartilage degradation was assessed by measurement of urinary levels of C-terminal crosslinking telopeptide of type II collagen (CTX-II, Cartilaps, Nordic Bioscience, Herlev, Denmark) [18]. The intra-assay and interassay coefficients of variation were lower than 10%.

### Evaluation of safety

Patient-reported adverse events (AEs) were recorded throughout the study. AEs were categorized using the Coding Symbols for Thesaurus of Adverse Reaction Terms (COSTART^®^) coding dictionary. Clinical laboratory measurements for safety monitoring were made throughout the study. Serious AEs were defined as any that resulted in death; were life threatening; resulted in hospitalization; resulted in persistent or significant disability or incapacity; or were judged to be medically significant.

Upper-GI AEs included the following symptoms and conditions: substernal chest pain; duodenitis; dyspepsia; dysphagia; oesophagitis; gastritis; bleeding gastritis; gastro-oesophageal reflux; oesophageal bleeding; GI bleeding; haematemesis; melena; abdominal pain; ulcers (duodenal, oesophageal, peptic, gastric); bleeding ulcers (duodenal, peptic, gastric); perforated ulcers (duodenal, peptic, gastric); perforated and bleeding ulcers (duodenal, peptic, gastric); and reactivated ulcers (duodenal, peptic, gastric).

### Statistical analysis

To ensure 80% power to detect a 20% effect of risedronate treatment versus placebo with respect to pain modification (quantified according to the WOMAC pain subscale, assuming a standard deviation of 70 mm on a 0- to 500-mm scale), a 1-year dropout rate of 20%, and a type I error rate of 5% without adjustment for two comparisons with placebo control, the sample size requirement was 100 patients per treatment group.

Analyses were undertaken on the intention-to-treat (ITT) population. This was defined as all randomized patients who received at least one dose of study medication. All statistical analyses were performed using a two-sided statistical test with a type-I-error rate of 0.05. Baseline characteristics were compared using Fisher's exact test for categorical variables and the Kruskal–Wallis test for continuous variables. Extended Mantel–Haenszel tests with pooled centres as strata were used for end points with categorical responses. Analysis of variance (ANOVA) methods were used. Symptom analyses were adjusted for baseline value (PGA < WOMAC total or subscale value, as appropriate), pooled study centres, baseline use of oestrogen or selective oestrogen receptor modulators, gender, age, body mass index, and baseline JSW. Mean JSW analyses were adjusted for pooled study centres, baseline use of oestrogen or selective oestrogen receptor modulators, gender, age, body mass index, and baseline JSW as covariates. Each risedronate group was compared with the placebo group. For walking aids, the percentages were compared with placebo using the Cochran–Mantel–Haenszel test after adjusting for pooled centres. Individual AEs and the proportion of clinically meaningful JSW progressors were analysed using Fisher's exact test.

The WOMAC scores were calculated in accordance with the WOMAC User's Guide [19]. The total scores were composed of subscales weighted as follows: pain = 42%, stiffness = 21%, and function = 37% [19]. For each subscale, the reported response was the patient's average. If at least two pain items, both stiffness items, or more than four physical function items were omitted, or if the patient's response was unclear, the items were regarded as invalid and the relevant subscale was not included.

## Results

### Patients

**Figure 1:**
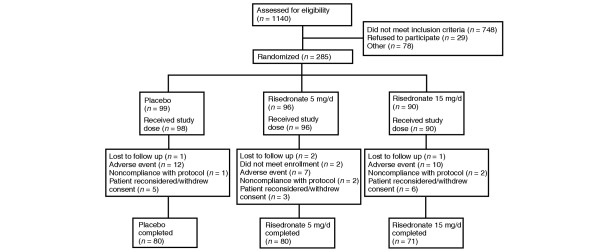
Disposition of patients with knee osteoarthritis in a controlled, randomized trial of risedronate.

Two hundred and eighty-five patients were considered eligible for the study and were randomized to treatment. Of these, 284 received at least one dose of study medication and were included in the ITT population, and 231 (81%) completed the study (placebo, *n *= 80; risedronate at 5 mg, *n *= 80; risedronate at 15 mg, *n *= 71) (Fig. 1). The number of patients who completed the study and the reasons for withdrawal were similar across treatment groups. Table 1 shows the baseline characteristics for the ITT population. These were similar between treatment groups; the average age of the patients was 63.3 years. There were no significant differences in the use of concomitant analgesics between treatment groups. Patients' compliance during study treatment, based on pill counts, was ≥ 83% and was comparable in the three treatment groups.

### Symptom outcome measures

**Figure 2:**
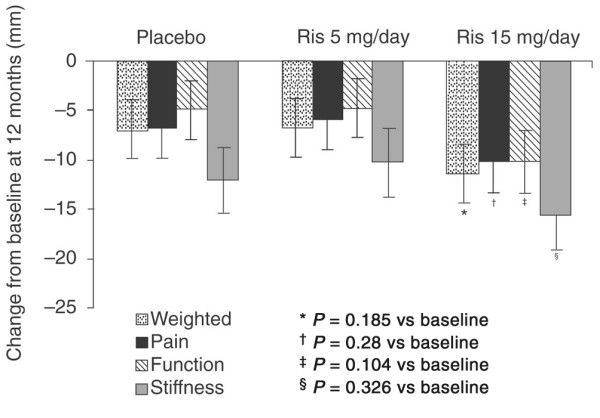
Changes in mean values at 12 months from baseline measures in patients with osteoarthritis. Patients were given risodronate (Ris) or placebo. Scores were the weighted composite of the 24 question scores on the visual analogue scale (1 to 100 mm) of the Western Ontario and McMaster Universities (WOMAC) osteoarthritis index or its subscales for pain, stiffness, and physical function. Vertical lines represent standard errors of the mean. *P *values refer to risedronate (15 mg) vs baseline values.

There was an improvement from baseline values in the symptom outcome measure of total WOMAC scores (weighted and unweighted) (unweighted not shown) and the subscales for all treatment groups (Fig. 2). The group given risedronate at 15 mg showed a trend towards improvement from baseline values, although the differences were not statistically significant (*P *values from 0.10 to 0.33).

**Figure 3:**
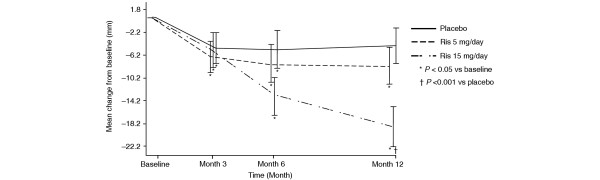
Changes in mean patient global assessment after risedronate or placebo in osteoarthritis. Vertical lines represent standard errors of the mean. Ris, risedronate.

Assessment of PGAs revealed a statistically significant improvement with risedronate at 15 mg compared with placebo at 1 year (-19.4 for risedronate at 15 mg versus -5.7 for placebo, *P *< 0.001) (Fig. 3). Although all treatment groups showed a significant improvement from baseline values at 3 months, the improvement in the group receiving risedronate at 15 mg continued to increase with time, whereas the level of improvement with placebo or risedronate at 5 mg did not show any further improvement after 6 months.

Analysis of the use of walking aids during study treatment showed a statistically significant difference in the proportion of patients who used walking aids in patients treated with risedronate at 15 mg (7 patients, 4% reduction) compared with placebo (21 patients, 8% increase) (*P *= 0.009) at 12 months compared with the proportion of patients that had reported using a walking aid during the previous year.

### Structure outcome measures

Assessment of the mean change from baseline values in minimum JSW in the medial compartment of the tibiofemoral joint at 1 year showed that there was a trend for patients receiving risedronate at 5 mg (JSW -0.08 ± 0.44 mm) or 15 mg (JSW -0.06 ± 0.25 mm). The change was greater in patients receiving placebo (JSW -0.12 ± 0.42 mm) compared with baseline values. Overall, the difference between treatment groups in loss of JSW at 12 months was not statistically significant (*P *= 0.275). The loss in JSW from baseline values was statistically significant only in the placebo group (*P *< 0.05).

In terms of detectable progression (i.e. loss of JSW ≥ 25% or ≥ 0.75 mm), the analysis of the distribution of change from baseline values in JSW at 1 year showed a greater presence of detectable progression in the placebo (8%) and risedronate (5 mg) (4%) (*P *= 0.36) groups than in the risedronate (15 mg) group (1%) (*P *= 0.067). The patients with JSW loss of >0.75 mm included none of the patients treated with risedronate at 15 mg and 6% of the patients treated with placebo (*P *= 0.060). Similarly, only 1% of the patients treated with risedronate at 15 mg but 7% of the patients treated with placebo had >25% loss of JSW (*P *= 0.12).

### Structure–symptom relation

**Figure 4:**
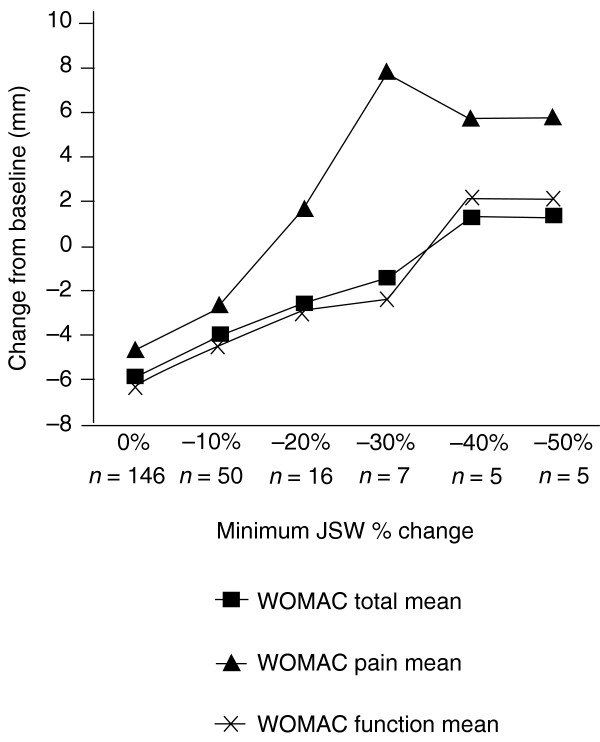
Relation between WOMAC scores and minimum percentage change in JSW after 1 year in patients with osteoarthritis. Scores were the weighted composite (1 to 100 mm) of the Western Ontario and McMaster Universities (WOMAC) osteoarthritis index or its subscales for pain and physical function. JSW, joint-space width.

Figure 4 shows the relation between structure and symptoms for this population of patients. The mean WOMAC total score and the scores on the pain and function subscales increased (i.e. symptom severity increased) with increasing loss of JSW. In the group of patients with any loss of JSW, the mean changes of the WOMAC total score and the scores on the pain and function subscales at 1 year were -5.9 mm, -4.6 mm, and -6.3 mm, respectively, indicating that these symptoms were not increasing overall. In contrast, for the subset of patients with a loss in JSW of 40% or more, the corresponding mean changes were +1.4 mm, +6.0 mm, and +2.3 mm, indicating increased symptom severity in these patients concurrent with narrowing of their knee-joint space.

### Markers of biochemical turnover

**Figure 5:**
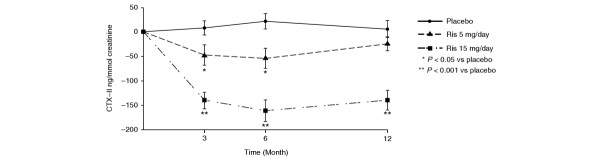
Changes in urinary CTX-II levels after treatment of osteoarthritis patients with placebo or risedronate. Ris, risedronate.

Risedronate treatment significantly reduced markers of cartilage degradation (Fig. 5) and bone resorption compared with placebo. At 1 year, treatment with risedronate at 15 mg significantly decreased mean urinary CTX-II values, by -22.8% ± 5.35; urinary NTX-I was reduced by -32.9% ± 4.92 relative to baseline values (*P *< 0.05). Dose-dependent effects were also observed with the 5-mg dose compared with placebo, but to a lesser magnitude. This finding is consistent with the known pharmacologic effect of risedronate on bone turnover. At 1 year, CTX-II and NTX-I values in the placebo group were significantly higher than those in the risedronate 15-mg group (14.5% ± 5.4 and 17.2% ± 4.9 higher, respectively). Significant decreases in bone alkaline phosphatase were observed in the risedronate groups compared with placebo. At 1 year, the mean decreases in the groups receiving risedronate at 15 mg and 5 mg were 29.1% ± 2.6 and 19.5% ± 2.5, respectively, compared with a mean decrease of 2.7% ± 2.5 in the placebo group (*P *< 0.001).

### Safety

The frequencies of AEs were similar in the two treatment groups (Table 2). There were no clinically meaningful differences between groups in the percentage of patients with AEs in any body system and there were no significant differences in routine clinical chemistry parameters between the risedronate groups and the placebo group. The numbers of patients who dropped out of the study because of AEs were similar. Overall, 34 patients reported a total of 53 serious AEs. Investigators considered four serious AEs as possibly related to study treatment; two of these (rash and diarrhea) were in patients treated with placebo and two (anaemia and increased general joint pain) were in patients treated with risedronate at 5 mg.

Table 2 provides a summary of adverse events for the ITT population and the frequency of the overall GI AEs and the most common upper-GI AEs. Forty-seven upper-GI events were reported in 38 patients, of which abdominal pain and dyspepsia were the most frequently reported. The majority of the upper-GI AEs occurred in patients with a history of GI disease; there were no significant differences between the groups given risedronate and the group given placebo in the incidence of upper-GI AEs in these patients.

## Discussion

Increased evidence of the role of bone in both the initiation and progression of OA has resulted in an interest in drugs that affect bone metabolism and might slow or even halt the process of joint degeneration [6]. The early findings reported here suggest that the bisphosphonate risedronate may have disease-modifying effects in patients with knee OA. A recent cross-sectional study also suggests an association between antiresorptive treatments (oestrogen or bisphosphonate) and improved symptoms and/or decreased bone marrow abnormalities [20].

Positive trends were observed with risedronate treatment with regard to symptomatic improvement, as assessed by the WOMAC index. Treatment with risedronate at 15 mg resulted in a consistent trend in improvement in WOMAC scores, whereas the group receiving placebo showed less improvement. The group receiving risedronate at 15 mg showed a significant improvement in the PGA of OA compared with placebo. Similarly, the percentage of patients who used a walking aid during the study decreased in the group treated with risedronate at 15 mg, contrasting with an increase in the placebo group. While the differences in JSW among the groups were not significant, there was a trend for less loss in the risedronate (15 mg) than in the placebo group. When the data were analysed in a post hoc manner to identify patients with detectable progression (i.e. approximately four times the precision of the measurement), 8% of patients receiving placebo and 1% of patients receiving 15 mg risedronate were found to exhibit this degree of progression. Additionally, risedronate significantly reduced levels of bone resorption and cartilage degradation, as assessed by NTX-I and CTX-II markers, respectively.

Despite these encouraging results, they were not confirmed in multicentre studies of risedronate treatment for 2 years using a similar protocol [21]. The following provides perspectives comparing the two studies. In our study, there was a dose-dependent trend for improvement in the WOMAC score. In the 2-year North American study, which enrolled 1,232 patients with knee OA, the placebo effect was approximately twice that observed in our study, and was comparable to the magnitude of change observed in the group given 15 mg risedronate in our study. Both studies showed significant decreases in CTX-II, the marker of cartilage degradation, but these were not associated with an attenuation of joint-space loss This lack of association may be related to the length of observation in these studies. Reijman and colleagues [22] observed 1,235 men and women with OA, followed up over an average period of 6.6 years. The subjects with baseline CTX-II levels in the highest quartile had a sixfold risk of progression of knee OA, defined as a decrease in JSW ≥ 2 mm, in comparison with subjects in the lowest quartile [22]. This suggests that an enriched population of subjects with an elevated rate of cartilage loss combined with a longer study period may be required in order to observe significant treatment effects.

Our study is one of the first to suggest a correlation between symptoms and structure in OA, although preliminary results with doxycycline in the treatment of obese women with knee OA have reported a significant reduction in the proportion of follow-up visits in which a clinically significant increase in pain occurred, favouring treatment over placebo, and coinciding with a decrease in joint-space narrowing [23]. This finding is important, because it runs contrary to previous results, which have suggested a poor correlation between these disease features [24,25]. The limitations of our study include the small number of patients (*n *= 10) with the greatest loss of JSW (>40%), for observations of concurrent increase in joint-space narrowing and WOMAC symptom severity scores. One possible explanation for discrepancies between the current study and previous studies is the difference in radiographic methodologies used. Several radiographic techniques have been described for measuring JSW in the knee. We used a highly standardized, fluoroscopic technique in which the knee was semiflexed. Recent studies have compared different radiograph imaging methods [26,27]. The results highlighted the importance of medial tibial plateau alignment with the central x-ray beam and showed that the standard clinical view of a standing extended knee is subject to considerable variability. In contrast, the fluoroscopic technique is well validated and is less variable in test-retest performance [15,28]. Further studies are required to further explore the possible correlation between symptoms and structure observed in our study. If validated, this relation may allow physicians to use the assessment of pain, perhaps in combination with a biomarker such as CTX-II, as a surrogate for other measures of disease progression

## Conclusion

This study is one of the first to show a correlation between symptoms and joint structure changes in knee OA. While our findings were suggestive of a beneficial effect of risedronate treatment on preservation of bone and cartilage, these trends seen in this study have not been observed in larger, multicountry cohorts.

## Abbreviations

AE = adverse event; BRISK = British Study of Risedronate in Structure and Symptoms of Knee OA; CTX-II = C-terminal crosslinking telopeptide of type II collagen; GI = gastrointestinal; ITT = intention-to-treat; JSW = joint-space width; NTX-I = N-terminal crosslinking telopeptide of type I collagen; OA = osteoarthritis; PGA = patient global assessment; VAS = visual analogue scale; WOMAC = Western Ontario and McMaster Universities [OA index].

## Competing interests

This manuscript was sponsored by a grant from Procter & Gamble.

## Authors' contributions

TDS, JFB, DJV, and JMM planned the study and prepared the manuscript. GAC performed the statistical analysis. JCB-W supervised radiological measurements. PG performed the marker assays. PGC assisted with the manuscript and recruited patients. All authors read and approved the final manuscript.

## Figures and Tables

**Table 1:** Baseline characteristics of the intention-to-treat (ITT) population with osteoarthritis of the knee

		Risedronate		
			
Characteristic	Placebo (*n *= 98)	5 mg/day (*n *= 96)	15 mg/day (*n *= 90)	*P*
Age (years)	63.2 (0.82)	62.9 (0.90)	63.8 (0.88)	0.652^a^
Height (cm)	164.3 (0.90)	165.3 (0.97)	165.1 (0.93)	0.614^a^
Body mass index (kg/m^2^)	29.2 (0.38)	29.0 (0.40)	29.2 (0.42)	0.799^a^
Sex (no. (%))				0.307^b^
Male	34 (35%)	40 (42%)	41 (46%)	
Female	64 (65%)	56 (58%)	49 (54%)	
Post-menopausal (no. (%))	54 (84%)	49 (88%)	43 (88%)	0.985^b^
Years since menopause	14.4 (1.22)	16.5 (1.57)	17.4 (1.42)	0.273^a^
Oestrogen or SERM use (no. (%))	18 (28%)	15 (27%)	10 (20%)	0.380^b^
Race (no.)				0.385^b^
Asian Oriental	1	0	0	
Asian Indian	1	0	2	
Caucasian	96	96	88	
Joint-space width (mm)	3.03 (0.05)	2.95 (0.05)	3.01 (0.06)	0.577^a^
WOMAC – weighted total score	50.3 (2.0)	46.1 (2.0)	49.4 (2.4)	0.281^a^
Use of walking aids (no. (%))	16 (16)	22 (23)	12 (13)	0.224^b^
uCTX-II (ng/mmol creatinine)	312.5 (19.9)	328.9 (29.7)	340.1 (24.0)	0.748^a^
uNTX-I (nmol/mmol creatinine)	40.3 (2.8)	42.3 (4.5)	38.6 (2.2)	0.954^a^

Unless indicated otherwise, values are means (standard errors of the mean). ^a^Kruskal–Wallis test; ^b^Fisher exact test. SERM, selective oestrogen receptor modulator; uCTX-II, urinary C-terminal crosslinking telopeptide of type II collagen; uNTX-I, urinary N-terminal crosslinking telopeptide of type I collagen; WOMAC, Western Ontario and McMaster Universities osteoarthritis index.

**Table 2:** Summary of adverse events (no. (%)) in patients with osteoarthritis who received risedronate or placebo

	Placebo	Risedronate	
	(*n *= 98)	5 mg/day (*n *= 96)	15 mg/day (*n *= 90)

Patients with AEs	94 (96%)	95 (99%)	84 (93%)
Dropouts due to AEs	12 (12%)	7 (7%)	10 (11%)
Overall GI AEs	15 (15%)	16 (17%)	7 (8%)
Abdominal pain	6 (6%)	7 (7%)	3 (3%)
Dyspepsia	7 (7%)	7 (7%)	3 (3%)
Gl disorder	3 (3%)	1 (1%)	1 (1%)

AE, adverse event; GI, gastrointestinal.
